# Long-Term Efficacy of Novel and Traditional Home-Based, Remote Inspiratory Muscle Training in COPD: A Randomized Controlled Trial

**DOI:** 10.3390/jcm14176099

**Published:** 2025-08-28

**Authors:** Filip Dosbaba, Martin Hartman, Magno F. Formiga, Daniela Vlazna, Jitka Mináriková, Marek Plutinsky, Kristian Brat, Jing Jing Su, Lawrence P. Cahalin, Ladislav Batalik

**Affiliations:** 1 Department of Physiotherapy and Rehabilitation, Faculty of Medicine, Masaryk University, 60200 Brno, Czech Republic; dosbaba.filip@fnbrno.cz (F.D.); hartman.martin@fnbrno.cz (M.H.); vlazna.daniela@fnbrno.cz (D.V.); minarikova.jitka@fnbrno.cz (J.M.); 2 Department of Rehabilitation, University Hospital Brno, 62500 Brno, Czech Republic; 3 Rehabilitation Clinic, Faculty of Medicine, Masaryk University, 60200 Brno, Czech Republic; plutinsky.marek@fnbrno.cz (M.P.); 60276@mail.muni.cz (K.B.); 4 Department of Rehabilitation and Sports Medicine, 2nd Faculty of Medicine, Charles University, University Hospital Motol, 150 06 Prague, Czech Republic; 5 Departamento de Fisioterapia, Faculdade de Medicina, Universidade Federal do Ceará, Fortaleza 60020-181, Brazil; magno.formiga@ufc.br; 6 Department of Respiratory Diseases, University Hospital Brno, 62500 Brno, Czech Republic; 7 School of Nursing, Tung Wah College, Hong Kong, China; jjsu@twc.edu.hk; 8 Department of Physical Therapy, University of Miami Miller School of Medicine, Coral Gables, FL 33146, USA; l.cahalin@miami.edu; 9 Department of Public Health, Faculty of Medicine, Masaryk University, 60200 Brno, Czech Republic

**Keywords:** chronic obstructive pulmonary disease, inspiratory muscle training, test of incremental respiratory endurance, home-based rehabilitation, adherence, maximal inspiratory pressure, sustained maximal inspiratory pressure

## Abstract

**Background:** Chronic obstructive pulmonary disease (COPD) is a progressive condition leading to declining lung function, dyspnea, and reduced quality of life. Pulmonary rehabilitation (PR) remains a cornerstone in COPD management; however, access remains limited, with less than 3% of eligible patients participating. Inspiratory muscle training (IMT), especially through novel methods like the Test of Incremental Respiratory Endurance (TIRE), offers a potential home-based alternative to traditional rehabilitation services. Despite growing interest, a key knowledge gap persists: few randomized trials have directly compared TIRE with threshold loading IMT over extended, largely unsupervised home-based periods while concurrently evaluating inspiratory muscle endurance and adherence. This randomized controlled trial aimed to evaluate the long-term efficacy of TIRE IMT compared to traditional threshold IMT and sham training in COPD patients. The study also assessed adherence to these home-based interventions, focusing on unsupervised periods without additional motivational support. **Methods:** A total of 52 COPD patients were randomly assigned to one of three groups: TIRE IMT, Threshold IMT, or Sham IMT. The study consisted of an 8-week supervised Phase I followed by a 24-week unsupervised Phase II. Training details: TIRE—session template set to 50% of the day’s maximal sustained effort; 6 levels × 6 inspirations (total 36) with preset inter-breath recoveries decreasing from 60 s to 10 s. Threshold IMT—spring-loaded valve set to 50% MIP (re-set at week 4); 36 inspirations completed within ≤30 min. Sham—valve set to minimal resistance (9 cmH_2_O); 36 inspirations within ≤30 min. Primary outcomes included changes in maximal inspiratory pressure (MIP) and sustained maximal inspiratory pressure. Secondary outcomes focused on adherence rates and correlations with functional capacity. **Results:** Of the 52 participants, 36 completed the study. Participant details: TIRE *n* = 12 (mean age 60.9 ± 12.9 years), Threshold *n* = 12 (67.4 ± 6.9 years), Sham *n* = 12 (67.3 ± 8.7 years); overall 21/36 (58%) men; mean BMI 30.0 ± 7.5 kg/m^2^. The TIRE IMT group demonstrated significantly greater improvements in MIP (31.7%) and SMIP compared to both the Threshold and Sham groups at 24 weeks (*p* < 0.05). Despite a decline in adherence during the unsupervised phase, the TIRE group maintained superior outcomes. No adverse events were reported during the intervention period. **Conclusions:** In this randomized trial, TIRE IMT was associated with greater improvements in inspiratory muscle performance than threshold and sham IMT. While adherence was higher in the TIRE group, it declined during the unsupervised phase. The clinical interpretation of these findings should consider the relatively wide confidence intervals and modest sample size. Nevertheless, the mean change in MIP in the TIRE arm exceeded a recently proposed minimal important difference for COPD, suggesting potential clinical relevance; however, no universally accepted minimal important difference exists yet for SMIP. Further adequately powered trials are warranted.

## 1. Introduction

Chronic obstructive pulmonary disease (COPD) is a prevalent and complex condition marked by a progressive and irreversible decline in lung function [1]. The global incidence of COPD is steadily increasing, leading to higher mortality and morbidity rates that greatly affect patients’ quality of life [2,3]. In the Czech Republic, an estimated 800,000 people, or 7–8% of the population, are living with COPD, underscoring the significant burden of this disease [4].

Pulmonary rehabilitation (PR) is a cornerstone therapy for COPD, shown to improve exercise tolerance, reduce dyspnea, and enhance quality of life [5]. Despite its benefits, less than 3% of eligible patients globally access PR services. Barriers include limited awareness among patients and providers, logistical challenges, inadequate insurance coverage, and inefficiencies in service delivery. Expanding access through alternative models, such as home-based or remote PR, could help bridge the gap, especially in underserved and rural areas [6,7].

In response to these challenges, home-based therapies, such as inspiratory muscle training (IMT), are gaining prominence for their ability to improve exercise capacity and reduce dyspnea in COPD patients [8]. The Test of Incremental Respiratory Endurance (TIRE) is a novel method for assessing and training inspiratory muscles and has recently been validated for use in COPD patients. TIRE provides comprehensive measures, including maximal inspiratory pressure (MIP), sustained maximal inspiratory pressure (SMIP), and inspiratory duration (ID), offering potential advantages over traditional IMT methods, which are often limited by workload capacity and duration of inspiration [9].

IMT has demonstrated significant clinical benefits in managing obstructive lung disease, making it a promising and accessible therapeutic option for COPD patients [10]. However, optimizing IMT methods to achieve the best functional outcomes remains critical. Traditional threshold loading IMT has notable limitations, including restricted workloads and inspiratory durations. In contrast, TIRE is designed to overcome these limitations by enabling patients to achieve higher inspiratory pressures throughout the full range of inspiration, making it a potentially more effective training tool [11,12,13].

In addition to their role as training outcomes, TIRE-derived measures such as SMIP, ID, and fatigue index test have been reported to correlate more strongly with COPD severity, dyspnea, and exercise capacity than traditional MIP [9,14]. These findings suggest that TIRE parameters may serve not only as sensitive markers of inspiratory muscle performance but also as potential surrogates for clinically relevant outcomes [8,15]. Therefore, our study also examined correlations between TIRE metrics and functional outcomes, aiming to explore their broader clinical significance.

The primary objective of this long-term randomized controlled trial is to evaluate the effectiveness of TIRE as a home-based IMT method in COPD patients, specifically focusing on training outcomes and patient adherence during the period from week 9 to week 24, when no additional motivational support was provided. It is hypothesized that TIRE will yield superior results compared to traditional threshold IMT protocols in terms of improving inspiratory muscle performance and COPD-related outcomes.

## 2. Materials and Methods

This study was a prospective, randomized controlled trial conducted in a clinical setting. The study protocol was approved by the local institutional medical research ethics committee (approval number 01-020420/EK, approval date 2 April 2020) and registered prior to patient enrollment with the Clinical Trial Registry (NCT04415788, https://www.clinicaltrials.gov/, accessed on 4 June 2020). All participants provided written informed consent before inclusion in the study.

The trial was conducted in collaboration with the Rehabilitation Department and the Department of Respiratory Diseases at University Hospital Brno. Between 7 May 2021, and 5 March 2024, patients with COPD were recruited and randomly assigned to one of three groups: two treatment groups and one sham intervention group, in a 1:1:1 ratio. Each participant underwent IMT, with training protocols delivered using one of two different devices, depending on group allocation.

The study consisted of two phases: an initial 8-week at-home training period with remote supervision (Phase I), followed by a 24-week unsupervised, independent respiratory training phase (Phase II). The study was conducted and reported following CONSORT guidelines, and the detailed design has been published elsewhere [12,13]. A completed CONSORT checklist has been provided as a Supplementary Table S1.

### 2.1. Study Population and Training Protocols

Participants were recruited from the outpatient clinics of the Department of Respiratory Diseases, University Hospital Brno. Eligibility required (i) adulthood with COPD confirmed clinically and by spirometry according to GOLD criteria (stages I–IV); (ii) the ability to independently use a computer, tablet, or smartphone and follow training instructions; (iii) clinical stability with no respiratory infection or COPD exacerbation in the preceding ≥2 months; and (iv) no participation in structured exercise programs during the previous 12 months.

Subjects were excluded if they had a history of lung surgery or lung cancer, or if they had any diagnosed cognitive impairment or dementia orthopedic, neurological, or neuromuscular disorders that could interfere with physical tests or participation in the study.

Eligible participants were randomized (1:1:1) to one of three home-based IMT regimens: (1) TIRE, (2) threshold-loading IMT, and (3) sham low-resistance training. All participants then completed daily, unsupervised IMT at home for 8 weeks. TIRE was delivered with the PrO_2_ system (Design Net, Smithfield, VA, USA; Supplementary Figure S1), whereas the threshold and sham protocols used the Threshold IMT trainer (Philips Respironics, Murrysville, PA, USA; Supplementary Figure S2). (Table 1 provides a summary of the distinctive training characteristics for each study group, and Supplementary File S1 contains a detailed description.

### 2.2. Training Procedure

In Phase I, each group of subjects was trained on their assigned procedures by a research team member, who is a registered physiotherapist, ensuring consistent instruction across all groups. To minimize bias and the risk of sample contamination, different team members were involved in the training sessions for each group, following standardized protocols. Following this initial instruction, subjects were expected to practice independently at home each day. Subjects were instructed to perform one training session per day, with each session consisting of 36 inspirations, as detailed in Table 1. The TIRE and traditional IMT protocols were based on previously published methods [9,12]. Regardless of the method used, subjects were required to fill out diary cards after each session, documenting both the number of breaths performed and the total number of sessions completed. This data was later used for compliance assessment. Participants also received detailed user guides developed by the research team, which included instructions on equipment setup, the training protocol, and contact information.

To support adherence, subjects from all three groups received weekly phone calls from a designated research team member. These calls were made to encourage compliance, address any questions or concerns, and collect information on symptoms and progress. To minimize bias during Phase I, different team members conducted calls for each group.

In Phase II (week 9 to week 24), all subjects were instructed to continue their training independently at home, without additional support or motivational phone calls. They were also asked to maintain diaries to record their daily training progress.

### 2.3. Study Outcomes

The primary outcome of the study was the change in inspiratory muscle strength and endurance, as measured by SMIP and MIP, in the TIRE IMT and Threshold IMT groups compared to the Sham IMT group. These parameters were assessed at baseline, at the end of Phase I (8 weeks), and at the end of the 24-week follow-up (Phase II) by the trained research assistant(s) who was(were) blinded to group allocation.

Secondary outcomes included the assessment of adherence to the training protocol across both phases of the study. Adherence was defined as the percentage of completed training sessions relative to the total number of planned sessions. Adherence rates were compared between Phase I (supervised) and Phase II (unsupervised).

Additionally, changes in the TIRE-specific parameters, including SMIP, MIP, inspiratory duration (ID), and force inspiratory time (FIT), were evaluated in both the TIRE IMT and Threshold IMT intervention groups. These measurements were taken at baseline, at the end of Phase I (8 weeks), and at the end of Phase II (24 weeks). The Sham IMT group was similarly assessed to determine if there were any changes in these parameters across both phases.

### 2.4. Sample Size

A power analysis was conducted based on findings from prior studies on TIRE metrics. Normative TIRE data showed effect sizes ranging from 0.50 to 1.00 when comparing SMIP across different age groups [14]. Additionally, a meta-analysis on inspiratory muscle strength reported a significant pooled effect size of 0.68 (95% CI 0.54–0.82; *p* = 0.001) for MIP [15]. Consequently, we estimated an effect size of approximately 0.75 as a reasonable assumption. Using the Portney and Watkins test, we determined that a minimum of 12 participants per group is required for statistical analysis, assuming 2 degrees of freedom, a power of 0.9, and an alpha level of 0.05. To account for potential exclusions or dropouts, new participants will be enrolled to ensure that each group comprises at least 12 subjects.

### 2.5. Randomization

A computerized randomization sequence was used to assign participants to one of three study groups. The process involved opaque, sealed, and sequentially numbered envelopes, ensuring allocation concealment until intervention assignment. While some healthcare providers were informed of group assignments, participants were instructed not to disclose this information to the physicians conducting the initial and final assessments, who remained blinded to minimize bias. The interventions were carefully designed to ensure similarity between groups, particularly the MTL and Sham MTL groups, to support blinding.

### 2.6. Statistical Analysis

The dataset included both continuous and categorical variables. Continuous variables were summarized as mean ± standard deviation, while categorical variables were reported as absolute and relative frequencies.

Spearman’s rank correlation coefficient was used to assess relationships between continuous variables, with statistical tests conducted for zero correlation. Differences between study groups at each visit were analyzed using parametric (ANOVA, Welch ANOVA) and non-parametric (Kruskal–Wallis) tests, with effect sizes calculated for each test.

To analyze changes within each group over time (between visits), marginal models using generalized least squares (GLS) were employed, which account for correlations between repeated measurements. Specifically, an autoregressive correlation structure of order 1 (AR(1)) was used to model the relationship between observations taken at different time points. This assumes that measurements closer in time are more strongly correlated, with the correlation decreasing as the time between measurements increases. Group, visit, and their interaction were treated as independent variables, and AR(1) correlation structures, along with different residual variance structures, were applied where necessary. A *p*-value of less than 0.05 was considered statistically significant. All analyses were performed using R software, version 4.3.1.

## 3. Results

**Figure 1:**
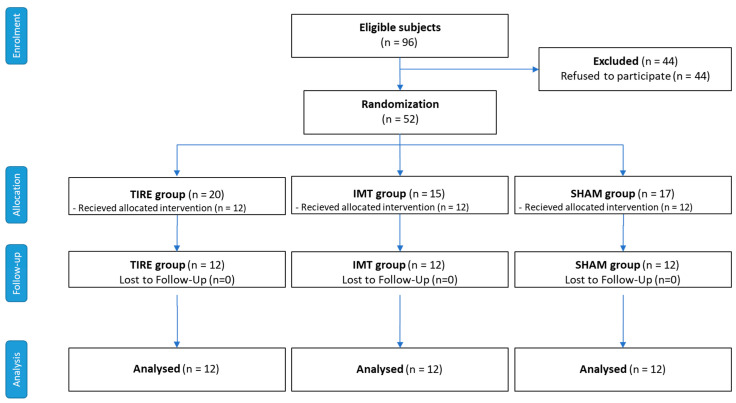
Flowchart of the study.

Out of 96 patients with stable COPD who were eligible, 52 patients agreed to participate, resulting in a participation rate of 54.2%. These patients were randomized into three intervention groups: TIRE IMT, Threshold IMT, and Sham IMT. Of these, 36 participants (69.2%) completed the 8-week intervention period (Phase I) and the subsequent 24-week follow-up period (Phase II). However, 16 participants (30.8%) discontinued their participation before the final assessment. The reasons for discontinuation were as follows: COPD exacerbations (9 participants, 17.3%), other health-related issues (4 participants, 7.7%), personal reasons (1 participant, 1.9%), missed required visit (1 participant, 1.9%), and technological issues (1 participant, 1.9%). These details are outlined in the CONSORT flow diagram (Figure 1). Despite these dropouts, the final analysis included 12 participants per group, as required by our power calculation. No adverse effects or COPD exacerbations directly related to IMT interventions were observed during the study.

Table 2 summarizes the baseline characteristics of the participants in each group. The average Charlson Comorbidity Index (CCI), used to estimate the presence of comorbidities and expected survival, was comparable across all groups (overall CCI of 4.28 ± 1.96). The mean age of the analysis cohort at 24 weeks was 65.2 ± 6.2 years, with 21 men (58%) represented. Only 4 patients reported being active smokers, and the mean BMI was 30 ± 7.48 kg/m^2^. Most patients in the analysis cohort (89%) had moderate COPD severity, classified as GOLD grades 2–3.

### 3.1. Effects

**Figure 2:**
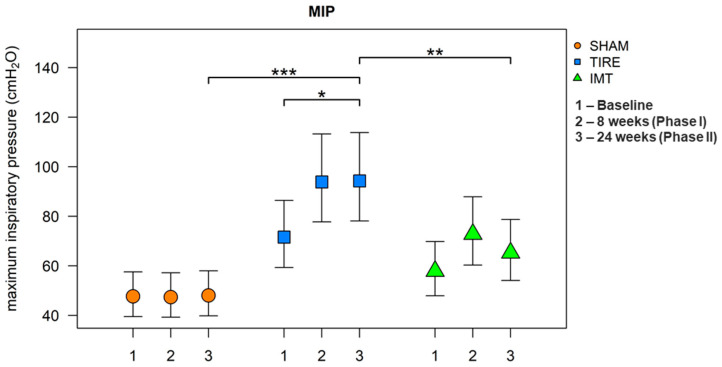
Comparison of MIP between and inside groups. Data are presented as adjusted difference (95% CI) between interventions. Symbols indicate significance levels (* *p* < 0.05, ** *p* < 0.01, *** *p* < 0.001) based on one-way ANOVA with post hoc pairwise comparisons.

**Figure 3:**
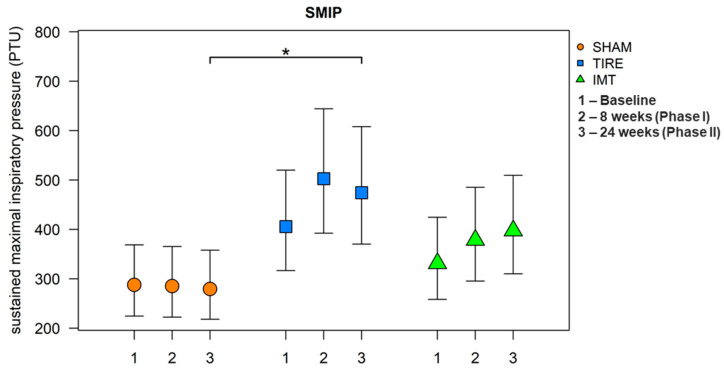
Comparison of SMIP between and inside groups. Data are presented as adjusted difference (95% CI) between interventions. Symbols indicate significance levels (* *p* < 0.05) based on one-way ANOVA with post hoc pairwise comparisons.

After 24 weeks, all parameters listed in Table 3 were evaluated. A large effect between groups was observed for the parameters MIP and SMIP. When comparing between groups, we observe a significant increase in these parameters in the TIRE IMT group compared to the SHAM IMT group. A significant increase in MIP and SMIP parameters is observed in both intervention groups, whereas no change in parameters describing inspiratory muscle endurance was observed in the SHAM control group. Although baseline and 24-week values of MIP and SMIP are already presented in Table 3, Figure 2 and Figure 3 were included to provide a clearer visualization of the temporal trends across all three measurement points and to illustrate between-group differences with corresponding confidence intervals.

It was found that the mean values of the MIP parameter were statistically significantly different between groups (ANOVA: F(2.33) = 11.99, *p* < 0.001, h2 = 0.42). Post hoc tests revealed significantly higher mean values in the TIRE IMT group compared to the SHAM IMT group (diff = 0.674, 95% CI: 0.336;1.013, *p* < 0.001) and in the TIRE IMT group compared to the Threshold IMT group (diff = 0.368, 95% CI: 0.030;0.706, *p* = 0.031). Marginally higher values of this parameter were observed in the Threshold IMT group compared to the SHAM IMT group. In the main intervention group, TIRE IMT, a significant increase in the MIP parameter value was observed between baseline and 24 weeks (diff = 0.276, 95% CI: 0.091;0.461, *p* = 0.003). Within the Threshold IMT group, the MIP value did not show a statistically significant increase between baseline and 24 weeks. Statistically significant differences in mean MIP values were found both between groups and between visits. The TIRE IMT group showed higher values compared to SHAM by an average of 50.0% (95% CI: 8.7;107.2, *p* = 0.013). At 24 weeks, the TIRE IMT group showed higher values on average by 44.5% (95% CI: 4.6;99.5, *p* = 0.024) compared to Threshold IMT. Compared to baseline, the 24-week visit in the TIRE IMT group showed an increase in MIP values by 31.7% on average (95% CI: 2.9;68.6, *p* = 0.030).

Statistically significant differences in mean SMIP values between groups were found: at 24 weeks, TIRE showed higher values compared to SHAM by a mean of 69.8% (95% CI: 11.0;159.6, *p* = 0.013). The mean values of the SMIP parameter were found to be statistically significantly different from each other (ANOVA: F(2.33) = 4.74, *p* = 0.016, h2 = 0.22). Using post hoc tests, significantly higher mean values were identified in the TIRE IMT group compared to the SHAM IMT group (diff = 0.529, 95% CI: 0.100;0.959, *p* = 0.013). On the original scale, this corresponds to a higher SMIP value in the TIRE IMT group compared to the SHAM IMT group by a mean of 69.8% (95% CI: 10.5;160.8). Other differences were not statistically significant.

### 3.2. Correlations

Correlation analyses were performed for outcome measures at 24 weeks. Significant associations were observed between SMIP and 6MWT (R = 0.81, *p* = 0.002) and between FIT and 6MWT (R = 0.80, *p* = 0.003) in the sham group. Moderate but non-significant correlations between TIRE parameters (SMIP, MIP, ID, FIT) and functional outcomes (6MWT, mMRC) were observed in the intervention groups. Analyses based on pre–post changes showed similar but weaker patterns.

### 3.3. Adherence to IMT

Adherence to IMT during Phase II at 24 weeks was 54%, a 31% decrease from Phase I, when each group was supervised by a dedicated physiotherapist. Adherence rates varied between groups: the highest adherence was in the TIRE IMT group (75%), followed by the Threshold IMT group (62%) and the SHAM IMT group (58%).

The number of training diaries submitted also differed between groups. The TIRE IMT group submitted 58% of diaries, the Threshold IMT group 47%, and the SHAM IMT group 32%. For the Threshold and SHAM IMT groups, adherence data were obtained retrospectively through patient interviews, while for the TIRE IMT group, the data were collected remotely using software that tracked IMT completion.

During final interviews, patients cited various reasons for not completing the training. The most common reasons included illness (40%), holidays or public holidays (24%), lack of motivation (18%), fatigue from COPD or the IMT itself (12%), and other reasons (6%).

## 4. Discussion

The present study assessed the long-term efficacy and adherence to novel (TIRE) and traditional IMT methods as home-based interventions for patients with COPD [10,16,17]. The findings suggest that TIRE IMT, a method specifically designed to improve both inspiratory muscle strength and endurance, offers superior outcomes compared to traditional threshold-based IMT and a sham control group. This is particularly evident in the significant improvements in key physiological parameters such as MIP and SMIP.

One of the primary findings of this study is that TIRE IMT resulted in greater improvements in MIP and SMIP compared to both the Threshold IMT and Sham IMT groups. These improvements were particularly noticeable at the 24-week follow-up, suggesting that the effects of TIRE IMT are not only robust but also sustainable over time. Specifically, the TIRE group demonstrated an increase in MIP of 31.7% from baseline, a result that was both statistically and clinically significant. These results are in line with those reported in Figueiredo et al. (2020), who demonstrated that isolated IMT significantly increased maximal inspiratory pressure, a measure that reflects the strength of the inspiratory muscles [18]. Improvements in maximal inspiratory pressure are crucial for COPD patients, as stronger inspiratory muscles help reduce breathlessness and enhance functional capacity, leading to better overall respiratory function and quality of life [19,20]. Furthermore, Shiraishi et al. (2024) also found that IMT significantly improved diaphragm excursion and exercise tolerance, supporting the superior functional outcomes observed in our TIRE IMT group [17].

In contrast, traditional threshold IMT, although showing moderate improvements in MIP and SMIP, did not perform as well as the TIRE method. This may be attributed to the limitations inherent in threshold loading IMT, such as its inability to fully engage inspiratory muscles throughout the entire range of motion. These findings highlight the potential for novel IMT methods, such as TIRE, to provide more comprehensive training, leading to better clinical outcomes. Similarly, O’Connor et al. (2019) showed that IMT was an acceptable treatment option for patients with COPD who declined traditional PR, offering a valuable alternative therapy [16].

Interestingly, the sham group showed no significant changes in MIP, SMIP, or other outcome measures, confirming that the observed effects in the intervention groups were due to the active training and not other confounding variables such as time or placebo effects. Nevertheless, correlation analyses revealed strong associations between SMIP or FIT and the 6MWT in the sham group at 24 weeks, despite the absence of training effects. This unexpected finding may be attributable to random variability in a small sample, or placebo-related behavioral influences, rather than a true physiological adaptation. It underscores the potential clinical relevance of TIRE-derived parameters, as their association with functional capacity emerged even in the absence of effective training.

The improvements in MIP and SMIP observed in the TIRE group have important clinical implications for the management of COPD. As inspiratory muscle strength is closely linked to exercise tolerance and dyspnea, enhancing these parameters can lead to significant improvements in patients’ quality of life [21]. Zanaboni et al. (2023) support this by demonstrating that home-based telerehabilitation, including IMT, can reduce hospitalizations and improve long-term health status [22]. Similarly, Aktan et al. (2024) reported significant gains in MIP and functional capacity in patients undergoing home-based IMT interventions, reinforcing the utility of such approaches in chronic respiratory conditions [23].

Despite the potential benefits, adherence remains a critical factor in the long-term success of IMT. Our study found that adherence rates declined during the unsupervised phase of the intervention, particularly in the Threshold and Sham groups. The decline in adherence could be attributed to various factors, including lack of motivation, COPD-related fatigue, and external influences such as holidays or illness. The TIRE group, however, demonstrated the highest adherence rates, which may be due to the more engaging nature of the training or the feedback provided by the TIRE device. This is consistent with findings from Galdiz et al. (2021), who found that adherence to telerehabilitation programs remained a challenge but that remote monitoring could help mitigate some of these issues [24]. Additionally, the fact that TIRE parameters remained significantly improved even after the unsupervised Phase II suggests that the TIRE method’s capacity to engage the full inspiratory cycle may contribute to sustained benefits, despite reduced adherence during later phases.

The higher adherence observed in the TIRE group may be explained by the more engaging nature of the device, including real-time feedback and structured guidance, which could have enhanced patient motivation. In contrast, threshold and sham IMT protocols were more monotonous and provided no visual reinforcement, which may have contributed to lower adherence. These differences in adherence likely played a role in the superior outcomes of the TIRE group. However, the fact that TIRE participants maintained significantly greater improvements even during the unsupervised phase indicates that the training modality itself also contributed importantly to the observed benefits.

The integration of technology, such as remote monitoring, and motivational strategies like telecoaching could potentially enhance long-term adherence [25]. Several studies, including ours, indicate that the greatest improvements in inspiratory muscle performance occurred during the initial supervised phase, emphasizing the importance of continuous support [18,26].

### Limitations and Future Directions

Several limitations should be considered when interpreting these findings. First, 44 out of 96 eligible patients refused to participate, resulting in a participation rate of only 54.2%. Additionally, this study had a dropout rate of 30.8% during the 24-week follow-up period, with 16 participants discontinuing after randomization. While the sample size was determined based on a priori power analysis to ensure sufficient statistical power (with a target of 12 participants per group), the rate of discontinuation underscores the challenges of long-term adherence in COPD patients undergoing home-based interventions. This highlights the need for further research on strategies to improve retention in this patient population and also introduces potential selection bias, as those who declined participation or dropped out may have systematically differed from those who completed the study, potentially influencing the outcomes.

Another limitation is the presence of a small number of active smokers (n = 4 in total; two in the threshold IMT group and two in the sham group). Although their distribution was balanced and unlikely to have systematically influenced the results, smoking status may affect respiratory performance and responsiveness to IMT. Larger studies should therefore consider stratification or subgroup analyses according to smoking status.

Moreover, adherence was self-reported through diaries, which may not fully capture true compliance. This limitation is particularly relevant for the Threshold and Sham groups, where adherence data were collected retrospectively through patient interviews, increasing the risk of recall bias. As highlighted, maintaining adherence in unsupervised settings is challenging, and future studies should incorporate more objective adherence measures, such as wearable technology or automated logging systems [16].

Future research could benefit from larger sample sizes and more sophisticated methods for tracking adherence, such as wearable technology or integrated feedback systems that automatically record training sessions. Exploring the impact of sustained motivational support or regular follow-up on long-term adherence may also offer valuable insights into maintaining patient engagement during unsupervised phases of home-based IMT interventions. It is anticipated that combining IMT with telehealth strategies could further improve adherence and enhance long-term outcomes, providing a more structured and supportive environment for patients.

## 5. Conclusions

This randomized trial found that TIRE IMT was associated with statistically significant improvements in inspiratory muscle performance compared with traditional threshold loading and sham IMT. Given the relatively wide confidence intervals and sample size constraints, these results should be interpreted with appropriate caution. Notably, the mean increase in MIP observed in the TIRE group exceeded a recently proposed minimal important difference for COPD which supports possible clinical relevance; however, this threshold may vary by disease severity and setting. For SMIP, no universally accepted minimal important difference has been established to date, so we refrain from making definitive claims of clinical significance for this metric. Adherence was higher in the TIRE group but declined during the unsupervised phase, underscoring the need for strategies to support long-term engagement. Overall, TIRE IMT appears to be a promising home-based option for patients with limited access to conventional PR, and larger, adequately powered studies should confirm these findings and define clinically meaningful thresholds for TIRE-derived outcomes.

## Figures and Tables

**Table 1:** Proposed training characteristics per study group [12].

Group	Device	Training Load	Training Volume	Training Frequency
TIRE IMT	PrO2	On-screen training template set at 50% of the subject’s MIP and SMIP.	Six levels (A–F) with 6 inspirations per level for up to 36 efforts per session. Pre-set recovery times between breaths: 60 s at level A to 50, 40, 30, 20 and 10 s at levels B to F, respectively.	1/day
Threshold IMT	Threshold	One-way spring-loaded valve set at 50% of the subject’s MIP.	36 inspirations performed using the device within a 30 min period.	1/day
Sham IMT	Threshold	One-way spring-loaded valve set to its minimal resistance (−9 cmH_2_O).	36 inspirations performed using the device within a 30 min period.	1/day

Abbreviations: MIP, maximal inspiratory pressure; cmH_2_O, centimeter of water column; SMIP, sustained maximal inspiratory pressure; TIRE, test of incremental respiratory endurance; IMT, inspiratory muscle training.

**Table 2:** Baseline characteristics.

	TIRE IMT	Threshold IMT	Sham IMT
Subjects, n (%)	12 (33)	12 (33)	12 (33)
Age (years)	60.92 ± 12.94	67.42 ± 6.86	67.25 ± 8.68
Gender (men), n (%)	7 (58)	7 (58)	7 (58)
BMI (kg/m^2^)	32.02 ± 9.43	29.58 ± 6.17	28.43 ± 6.32
Active smoker, n (%)	0 (0)	2 (17)	2 (17)
GOLD grade (A/B/C/D) %	1/33/50/16	0/42/50/8	0/33/58/9
Charlson index	4.08 ± 2.19	4.5 ± 1.78	4.25 ± 2.18
Cardiovascular disease, n (%)	5 (42)	6 (50)	4 (33)
Diabetes mellitus, n (%)	2 (17)	2 (17)	3 (25)
Hypertension, n (%)	4 (33)	0 (0)	4 (33)
Obstructive sleep apnea, n (%)	1 (8)	1 (8)	2 (17)
Hyperlipoproteinemia, n (%)	2 (17)	2 (17)	3 (25)

Abbreviations: IMT, inspiratory muscle training; TIRE, test of incremental respiratory endurance; n, number of patients; BMI, Body Mass Index; GOLD, Global Initiative for Chronic Obstructive Lung Disease (GOLD).

**Table 3:** Study outcomes.

Outcomes	TIRE IMT		Threshold IMT		Sham IMT		Between Groups *p* Value
	Baseline	24 Week	Baseline	24 Week	Baseline	24 Week	
MIP (cmH_2_O)	72.67 ± 13.16	97.75 ± 29.12 *	60.17 ± 16.76	69.33 ± 24.54	50.58 ± 17.63	50.67 ± 16.54	<0.001
SMIP (PTU)	441.58 ± 185.56	534.25 ± 292.71	349.92 ± 117.29	419.67 ± 158	311.67 ± 126.11	303.83 ± 124.79	0.015
ID (s)	11.43 ± 4.57	13.08 ± 4.21	10.38 ± 2.49	11.56 ± 3.02	10.96 ± 3.18	10.03 ± 3.11	0.115
FIT (value)	17.98 ± 13.24	24.13 ± 19.14	12.83 ± 6.48	16.54 ± 9.72	12.3 ± 7.71	14.18 ± 7.88	0.280
FEV1 (L)	1.55 ± 0.66	1.49 ± 0.73	1.11 ±0.32	1.10 ± 0.27	1.35 ± 0.64	1.41 ± 0.63	0.134
FVC (L)	2.47 ± 0.87	2.46 ± 0.95	2.23 ± 0.47	2.28 ± 0.51	2.41 ± 0.99	2.41 ± 1.08	0.871
6MWT (m)	382.17 ± 90.34	423 ± 120.83	367 ± 88.76	405.5 ± 82.25	359.67 ± 118.09	356.42 ± 115.84	0.309
mMRC (value)	1.83 ± 1.11	1.75 ± 1.14	2 ± 1.21	1.83 ± 1.27	1.83 ± 1.40	1.58 ± 1	0.927
BODE (value)	2.67 ± 1.92	2.92 ± 1.93	3.58 ± 1.73	3.42 ± 1.83	2.42 ± 2.11	2.33 ± 2.06	0.356

Abbreviations: * Statistically significant values; IMT, inspiratory muscle training; TIRE, test of incremental respiratory endurance; m, month; MIP, maximal inspiratory pressure; cmH_2_O, centimeter of water column; SMIP, sustained maximal inspiratory pressure; PTU, pressure time unit; ID, inspiratory duration; s, second; FIT, fatigue index test; FEV1, one second forced expiratory volume; L, liter; FVC, forced vital capacity; 6MWT, six minute walking test; mMRC, modified medical research council; BODE, index.

## Data Availability

The datasets used and/or analyzed during the current study available from the corresponding author upon reasonable request.

## Supplementary Materials

The following supporting information can be downloaded at: https://www.mdpi.com/article/10.3390/jcm14176099/s1, Figure S1: PrO2 device; Figure S2: Threshold IMT; Table S1: Checklist; File S1: Threshold IMT.

## Abbreviations

The following abbreviations are used in this manuscript:

BMI	Body Mass Index
BODE	Body mass index, airflow Obstruction, Dyspnea, and Exercise capacity index
CCI	Charlson Comorbidity Index
CI	Confidence Interval
cmH_2_O	Centimeter of water column
CONSORT	Consolidated Standards of Reporting Trials
COPD	Chronic Obstructive Pulmonary Disease
FEV1	Forced Expiratory Volume in one second
FVC	Forced Vital Capacity
GOLD	Global Initiative for Chronic Obstructive Lung Disease
GLS	Generalized Least Squares
ID	Inspiratory Duration
IMT	Inspiratory Muscle Training
mMRC	modified Medical Research Council
MIP	Maximal Inspiratory Pressure
PR	Pulmonary Rehabilitation
PTU	Pressure Time Unit
RV	Residual Volume
SMIP	Sustained Maximal Inspiratory Pressure
TIRE	Test of Incremental Respiratory Endurance
TLC	Total Lung Capacity
6MWT	Six-Minute Walking Test
