# Supplementary material for: Long-Term Efficacy of Novel and Traditional Home-Based, Remote Inspiratory Muscle Training in COPD: A Randomized Controlled Trial
Source: J Clin Med. 2025 Aug 28;14(17):6099. doi: 10.3390/jcm14176099 (PMC12429686; doi:10.3390/jcm14176099)
Supplement: Supplementary file 1 [file jcm-14-06099-s001.zip › jcm-3837798-supplementary.pdf]

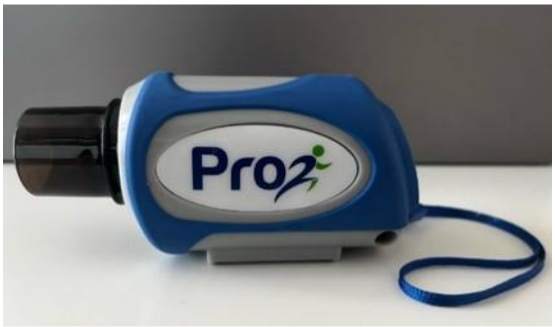

Supplementary Figure 1 PrO2 device

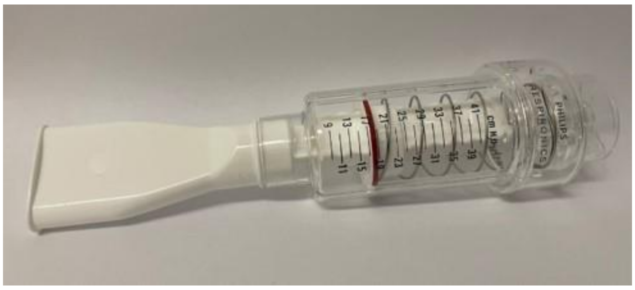

Supplementary Figure 2 Threshold IMT
Supplementary File 1

Table S1

Section/topic	No	CONSORT 2025 checklist item description	Reported on page no.
**Title and abstract**			
Title and structured abstract	1a	Identification as a randomised trial	1
	1b	Structured summary of the trial design, methods, results, and conclusions	1,2
**Open science**			
Trial registration	2	Name of trial registry, identifying number (with URL) and date of registration	1,4
Protocol and statistical analysis plan	3	Where the trial protocol and statistical analysis plan can be accessed	4
Data sharing	4	Where and how the individual de-identified participant data (including data dictionary), statistical code and any other materials can be accessed	13
Funding and conflicts of interest	5a	Sources of funding and other support (eg, supply of drugs), and role of funders in the design, conduct, analysis and reporting of the trial	13
	5b	Financial and other conflicts of interest of the manuscript authors	13
**Introduction**			
Background and rationale	6	Scientific background and rationale	3,4
Objectives	7	Specific objectives related to benefits and harms	3,4
**Methods**			
Patient and public involvement	8	Details of patient or public involvement in the design, conduct and reporting of the trial	NA
Trial design	9	Description of trial design including type of trial (eg, parallel group, crossover), allocation ratio, and framework (eg, superiority, equivalence, non-inferiority, exploratory)	4
Changes to trial protocol	10	Important changes to the trial after it commenced including any outcomes or analyses that were not prespecified, with reason	NA
Trial setting	11	Settings (eg, community, hospital) and locations (eg, countries, sites) where the trial was conducted	4,5
Eligibility criteria	12a	Eligibility criteria for participants	4,5
	12b	If applicable, eligibility criteria for sites and for individuals delivering the interventions (eg, surgeons, physiotherapists)	4,5
Intervention and comparator	13	Intervention and comparator with sufficient details to allow replication. If relevant, where additional materials describing the intervention and comparator (eg, intervention manual) can be accessed	5
Outcomes	14	Prespecified primary and secondary outcomes, including the specific measurement variable (eg, systolic blood pressure), analysis metric (eg, change from baseline, final value, time to event), method of aggregation (eg, median, proportion), and time point for each outcome	5,6
Harms	15	How harms were defined and assessed (eg, systematically, non-systematically)	NA

Sample size	16a	How sample size was determined, including all assumptions supporting the sample size calculation	4
	16b	Explanation of any interim analyses and stopping guidelines	NA
Randomisation:			
Sequence generation	17a	Who generated the random allocation sequence and the method used	4
	17b	Type of randomisation and details of any restriction (eg, stratification, blocking and block size)	5
			**Reported on page no.**
Allocation concealment mechanism	18	Mechanism used to implement the random allocation sequence (eg, central computer/telephone; sequentially numbered, opaque, sealed containers), describing any steps to conceal the sequence until interventions were assigned	5,6
Implementation	19	Whether the personnel who enrolled and those who assigned participants to the interventions had access to the random allocation sequence	4
Blinding	20a	Who was blinded after assignment to interventions (eg, participants, care providers, outcome assessors, data analysts)	6
	20b	If blinded, how blinding was achieved and description of the similarity of interventions	NA
Statistical methods	21a	Statistical methods used to compare groups for primary and secondary outcomes, including harms	6
	21b	Definition of who is included in each analysis (eg, all randomised participants), and in which group	NA
	21c	How missing data were handled in the analysis	NA
	21d	Methods for any additional analyses (eg, subgroup and sensitivity analyses), distinguishing prespecified from post hoc	NA
**Results**			
Participant flow, including flow diagram	22a	For each group, the numbers of participants who were randomly assigned, received intended intervention, and were analysed for the primary outcome	7
	22b	For each group, losses and exclusions after randomisation, together with reasons	7
Recruitment	23a	Dates defining the periods of recruitment and follow-up for outcomes of benefits and harms	7
	23b	If relevant, why the trial ended or was stopped	NA
Intervention and comparator delivery	24a	Intervention and comparator as they were actually administered (eg, where appropriate, who delivered the intervention/comparator, how participants adhered, whether they were delivered as intended (fidelity))	7-9
	24b	Concomitant care received during the trial for each group	NA
Baseline data	25	A table showing baseline demographic and clinical characteristics for each group	7
Numbers analysed, outcomes and estimation	26	For each primary and secondary outcome, by group:<br>● the number of participants included in the analysis<br>● the number of participants with available data at the outcome time point<br>● result for each group, and the estimated effect size and its precision (such as 95% confidence interval)<br>● for binary outcomes, presentation of both absolute and relative effect size	7-9
Harms	27	All harms or unintended events in each group	7

Ancillary analyses	28	Any other analyses performed, including subgroup and sensitivity analyses, distinguishing pre-specified from post hoc	NA
**Discussion**			
Interpretation	29	Interpretation consistent with results, balancing benefits and harms, and considering other relevant evidence	9-11
Limitations	30	Trial limitations, addressing sources of potential bias, imprecision, generalisability, and, if relevant, multiplicity of analyses	11

Group 1 – TIRE IMT

The TIRE training regimen involved the use of a tablet provided to the assigned subjects with pre-installed training software, along with a PrO2 device for the exercises. The software allowed subjects to track their inspiratory muscle performance via real-time graphical representations as their training progressed. Training consisted of six levels (A–F), with six inspirations at each level, totaling up to 36 breaths per session. Pre-set recovery times between breaths decreased as subjects advanced from 60 seconds at level A to 50, 40, 30, 20, and finally 10 seconds at level F [12].

All TIRE data were stored on the tablet and automatically synced to a cloud-based online platform for subsequent data retrieval and analysis. Each participant received a unique login and password, and the research team had access to the accounts for compliance tracking. This feature enabled the TIRE system to monitor adherence to the study protocol remotely [12].

Before each training session, subjects completed one maximal and sustained inspiratory effort, which set the baseline for that day's training. This effort was recorded by the software and displayed on the tablet as a training template set at 50% of the maximal effort. Subjects were required to match or exceed the on-screen template, achieving at least 90% of the redrawn maximal inspiratory pressure (MIP) and sustained maximal inspiratory pressure (SMIP). Although matching the 50% template allowed continuation, subjects were encouraged to perform maximal maneuvers from residual volume (RV) to total lung capacity (TLC), exceeding the required effort whenever possible [12].

After each successful breath, a countdown clock was triggered, and the subject waited for the pre-set recovery time before performing the next breath. If subjects failed to reach 90% of the MIP and SMIP for two consecutive breaths, they were prompted to either terminate or continue the session. It was important to note that subjects were instructed not to exhale into the device, using it only when prompted by the software to inhale and perform the required sustained effort [12].

Group 2 – Threshold IMT

Subjects assigned to the threshold loading training regimen received the traditional Threshold Inspiratory Muscle Trainer (Threshold IMT; Philips Respironics, USA). This device featured a one-way, spring-loaded valve at one end and a mouthpiece at the other, requiring subjects to inhale forcefully enough to overcome the valve's resistance, allowing proper inspiration to occur. In this setup, airflow was blocked until the subject generated sufficient inspiratory pressure to exceed the device's pre-set resistance in cmH2O.

The resistance was initially set to 50% of the subject's MIP at enrollment and was readjusted at week 4 to reflect 50% of their inspiratory muscle strength at that time. Subjects were coached to perform up to 36 breaths daily using the device and were instructed to complete each training session within a 30-minute period [12]. No fixed rest interval was prescribed; participants performed inspirations at

their own pace, with the requirement to complete 36 breaths within the allotted time.

Group 3 – Sham IMT (Low Resistance)

The Sham (Low Resistance) training regimen followed the same protocol as the threshold loading IMT, except that the device's resistance was set to its minimal level, 9 cmH2O. Subjects were instructed to perform up to 36 breaths daily using the device, with each session lasting no more than 30 minutes [12]. As in the threshold group, no controlled rest interval was used, and subjects paced their own breathing efforts across the session.
